# Daily Saline Nasal Douching for Chronic Allergic Rhinosinusitis: An Autobiographical Case Report

**DOI:** 10.7759/cureus.21153

**Published:** 2022-01-12

**Authors:** Maheshwar Lakkireddy

**Affiliations:** 1 Department of Orthopaedics, All India Institute of Medical Sciences, Bibinagar, Hyderabad, IND

**Keywords:** saline nasal irrigation, fess, fungal sinusitis, rhinosinusitis, allergy

## Abstract

Chronic allergic rhinosinusitis is a debilitating condition in a group of susceptible individuals with multifactorial etiology. I present my case of suffering from chronic allergic rhinosinusitis for 25 years treated with multiple drugs and surgeries with no relief. Adjunctive daily saline nasal douching has provided significant relief for three years to date. Genetic susceptibility with defective ciliary clearance, mucosal metaplasia with increased goblet cells secreting thick and inspissated mucus blocking the normal drainage, and stasis of secretions with inadequate mucosal immunity predisposes to polymicrobial infections, including fungal infections, antimicrobial resistance, and recurrent episodes of rhinosinusitis. Medical and surgical strategies often fail to manage this condition appropriately in a few cases. Adjunctive daily saline nasal douching is a safe and effective alternative to manage the debilitating symptoms of chronic allergic rhinosinusitis.

## Introduction

Chronic allergic rhinosinusitis is a debilitating condition in a group of susceptible individuals with multifactorial etiology [1-3]. Many of the affected individuals are young and suffer for a long time because of the recurrent nature of the disease. Failure to identify the exact cause and appropriately treat the underlying pathology leads to a protracted course of illness [3-5]. Long-term utilization of multiple drugs to symptomatically treat the presenting condition often leads to antimicrobial resistance and tachyphylaxis [6-9]. Polymicrobial colonization due to repeated infections and fungal sinusitis may complicate the later stages of the disease [5,7,10]. Aggressive surgical procedures performed for the same disturb the functional equilibrium of the nasal mucosa [5,11,12]. Feasible non-invasive interventions to halt the disease process will be very valuable for suffering individuals [13,14]. Here, I present my testimony of successfully managing the symptoms arising out of recurrent allergic chronic rhinosinusitis with daily saline nasal douching after having suffered from the condition for over 25 years with no success.

## Case presentation

**Figure 1:**
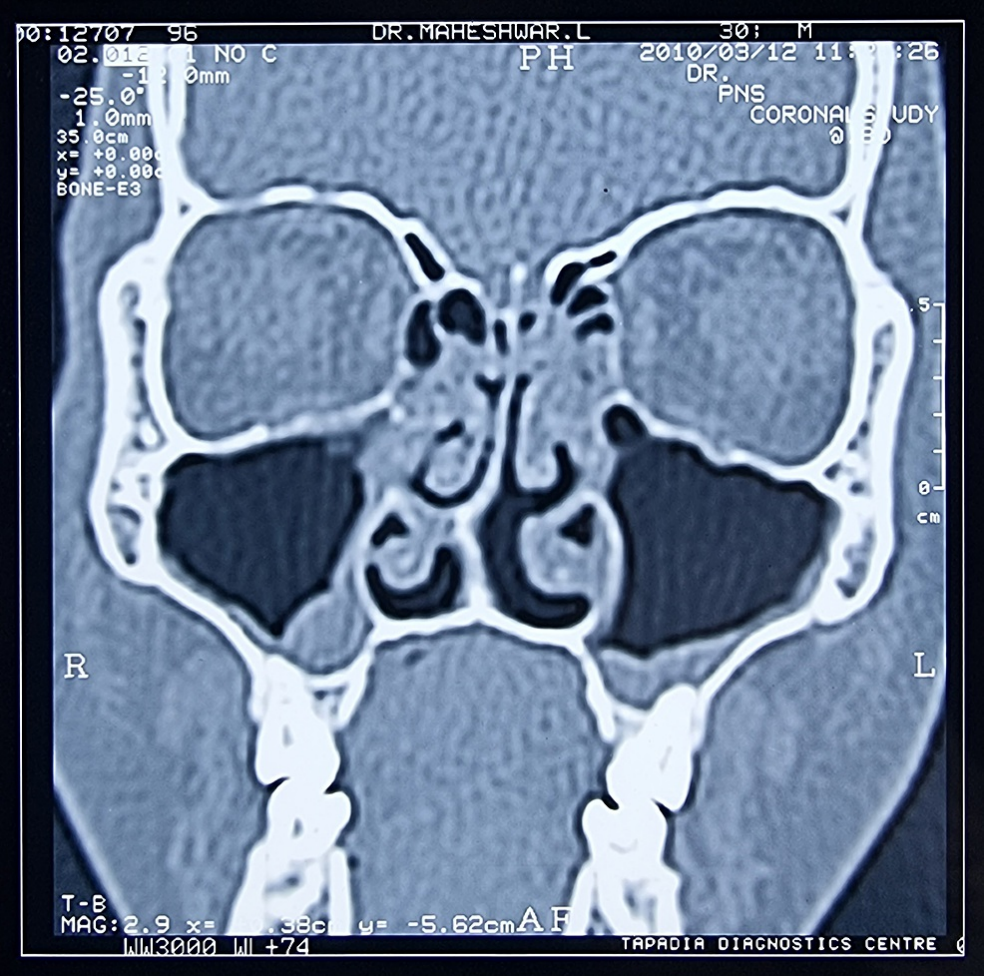
Coronal CT image of paranasal sinuses done in 2010 showing a deviated nasal septal spur with pansinusitis and blockade of the bilateral osteomeatal complex. CT: computed tomography

**Figure 2:**
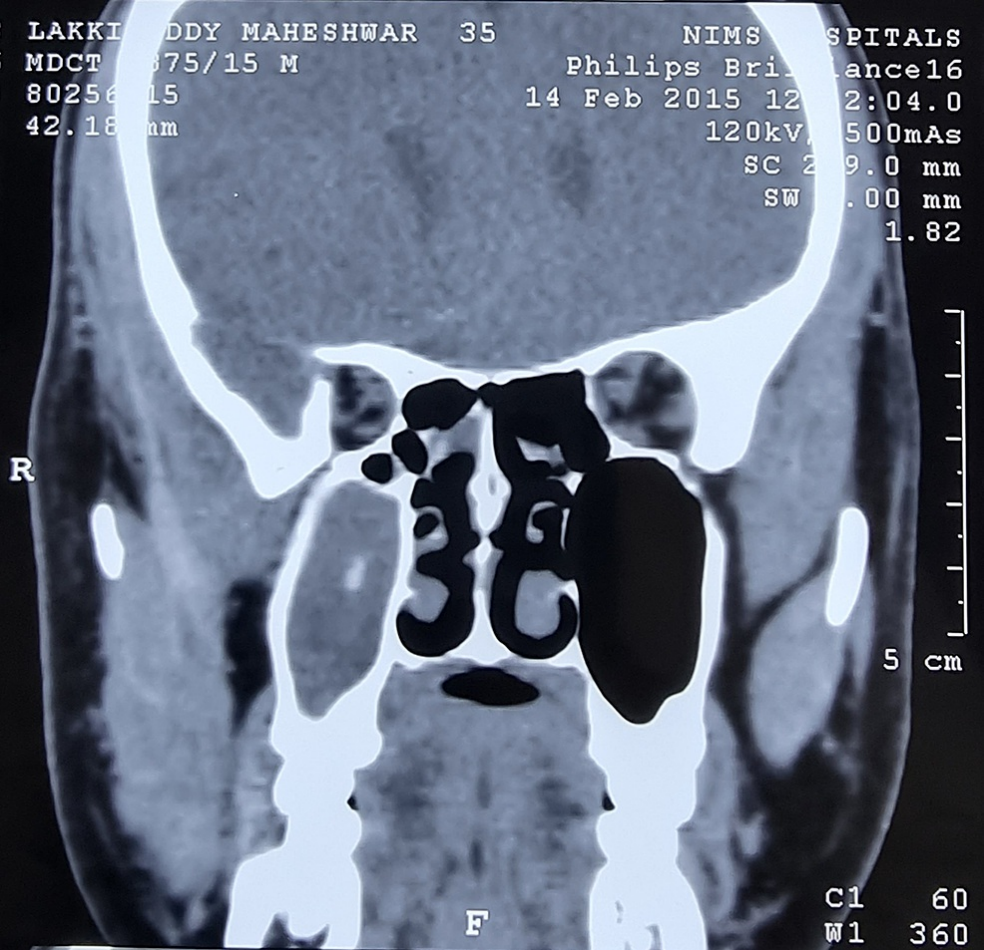
Coronal CT image of paranasal sinuses done in 2015 showing postoperative status with pansinusitis and significant mucosal thickening of the right maxillary sinus and double density sign signifying fungal sinusitis in the right maxillary sinus. CT: computed tomography

**Figure 3:**
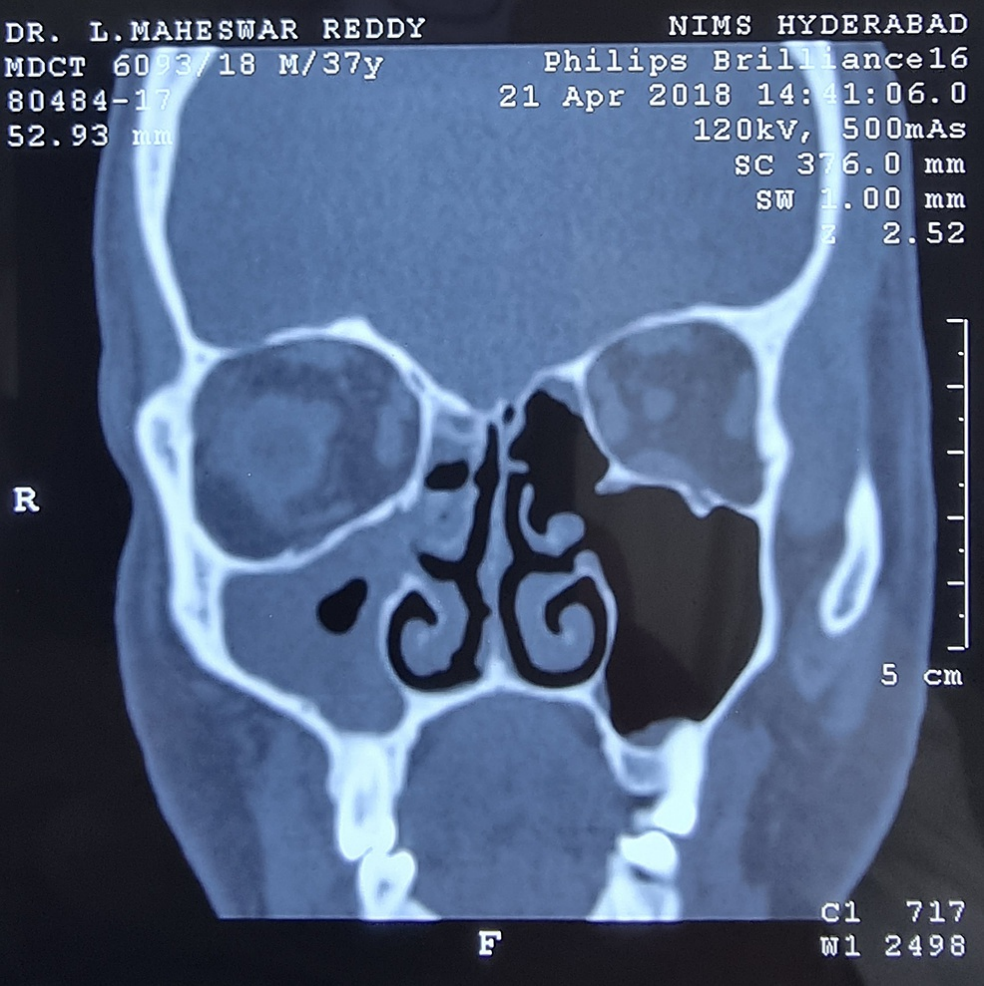
Coronal CT image of paranasal sinuses done in 2018 showing postoperative status with pansinusitis with significant mucosal thickening and collection of pus in the right maxillary sinus. CT: computed tomography

**Figure 4:**
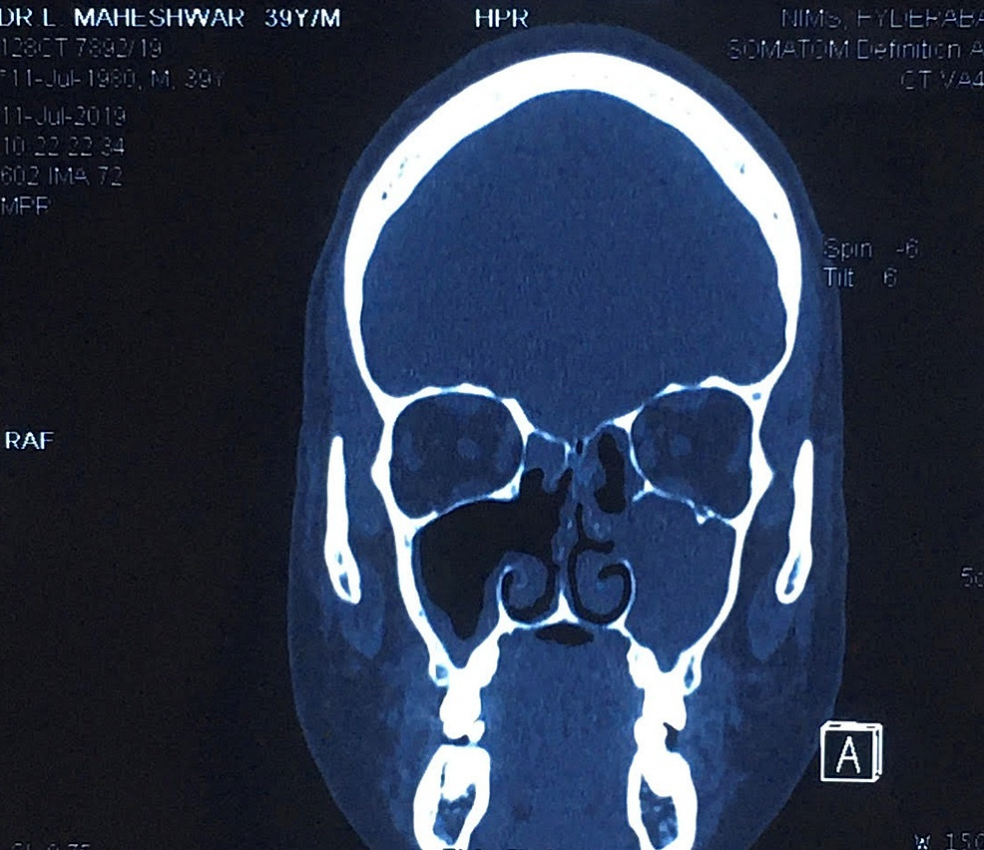
Coronal CT image of paranasal sinuses done in 2019 showing postoperative status with pansinusitis with bilateral mucosal thickening and collection of pus in the left maxillary sinus. CT: computed tomography

I had suffered from repeated episodes of sore throat at the age of 10 years. It was diagnosed as chronic suppurative tonsillitis, for which bilateral adenotonsillectomy was performed. Three years later and for the next five years, I suffered multiple episodes of nasal blockade, frontal headaches, and retroorbital pain. It was diagnosed as chronic pansinusitis and was treated with multiple cycles of antibiotics, oral and nasal decongestants, analgesics, and multiple antral lavages under local anesthesia. Although there was transient subsidence of symptoms, recurrence of the painful episodes could not be prevented. Therefore, I resorted to alternative therapies such as Ayurveda and homeopathy for allergic rhinitis and sinusitis. They too had no good effect in preventing the recurrent episodes. I underwent diagnostic nasal endoscopy for the first time in 2000 along with skin prick tests for the diagnosis of allergic rhinitis. I was found to be allergic to multiple allergens such as milk, meat, and banana. I had to avoid most of my routine foods to control my symptoms. I was relatively symptom-free for two years after avoiding most of the foods I was allergic to. Later on, I suffered from recurrent upper respiratory infections and multiple episodes of sinusitis for eight years. I was diagnosed with a deviated nasal septum and chronic pansinusitis in 2010 (Figure 1) and underwent functional endoscopic sinus surgery (FESS) with bilateral inferior reduction turbinectomy and enlargement of the osteomeatal complex. I was relatively symptom-free for two years but had recurrent and frequent episodes of sinusitis once again. As the symptoms were perceived to be due to the functional hypertrophy of the nasal mucosa in the absence of anatomical blockade owing to the previous surgery, I was prescribed fluticasone propionate 50 µg plus azelastine 140 µg combination nasal spray. Having used the nasal spray regularly, I had good symptom relief for two years. Because of the dryness of the nasal mucosa, I had to stop the spray after two years. One month later, I suffered a severe episode of pansinusitis which was resistant to higher antibiotics. Although I could somehow manage for another year with multiple medications, it became intolerable later on (Figure 2) and I had to undergo repeat FESS. Intraoperatively, a fungal ball was detected along with thickened mucosa. Fungal culture of the same yielded Zygomycetes. I was relatively asymptomatic for one year after a repeat FESS but again started to have recurrent episodes of resistant sinusitis (Figure 3). Following dengue fever, I had a severe episode of intolerable sinusitis (Figure 4). Fungal cultures of nasal and throat swabs and nasal discharge yielded *Aspergillus flavus*. I was advised to undergo FESS for the third time along with antifungals (Table 1). I declined the redo surgical option and opted for daily saline nasal douching along with the routinely used antihistamines prescribed by one of the ENT surgeons after a detailed examination. The nasal douching solution was prepared at home by mixing 200 mL of boiled and cooled drinking water in a squeeze bottle with 20 g of premixed sterile sodium chloride powder with xylitol to make a solution of 2% sodium chloride. Daily nasal saline douching performed once daily worked as an effective means to ameliorate my symptoms (nasal stuffiness, postnasal drip, nasal discharge, headache, fever episodes due to sinusitis, facial and retroorbital pain, etc.) in conjunction with routine antihistamines, which did not have much effect in the past 25 years. I have started using nasal douching regularly now for three years with utmost success and without a single episode of sinusitis again to date.

**Table 1:** A timeline of various events in chronological order. FESS: functional endoscopic sinus surgery

Year	Event	Intervention
1990	Chronic suppurative tonsillitis	Adenotonsillectomy
1994–1998	Recurrent sinusitis	Multiple antral lavages (6–8)
2000	Diagnosis of allergic rhinosinusitis	Diagnostic nasal endoscopy
2002–2010	Recurrent episodes of sinusitis	Medical management
2010	Chronic suppurative sinusitis	Septoplasty and FESS
2012–2014	Postoperative recurrence of sinusitis	Intranasal fluticasone usage
2015	Recurrence of severe sinusitis after stopping intranasal fluticasone	Redo FESS and biopsy (Zygomycetes detected)
2016–2019	Recurrent episodes of sinusitis	Symptomatic medical management
2019	Fungal rhinosinusitis (*Aspergillus*)	Antifungal therapy
2019–2021	Symptom-free interval	Daily saline nasal douching

## Discussion

Sinusitis is an acute inflammatory condition of the paranasal sinuses caused by microbial infection. Bacterial infections of the nasal cavity and paranasal sinuses are common in susceptible individuals [2]. Innate mucosal immunity, effective ciliary motility, and clearance are important factors for the early resolution of sinusitis [5,6,15]. Antibacterial agents for acute bacterial sinusitis fulfilling the treatment criteria, analgesics for pain, and local and systemic decongestants are commonly used drugs to treat the symptoms of sinusitis [9]. In genetically susceptible individuals, nasal and sinus mucosa reacts adversely to environmental factors such as dust, pollen, and some food materials and becomes metaplastic with thickening of the mucosa, increased number of goblet cells secreting aberrant mucus in excessive quantities leading to inspissation, and blockade of clearance pathways. Blockade leads to an increased propensity for microbial colonization and infection of the stagnated material [5,13,16]. Steroid nasal sprays are commonly used to treat such cases of allergic rhinitis and sinusitis. At times, they may decrease the local immunity and aid in the colonization of pathogenic fungi over long-term utilization [5,8,10]. In some genetically susceptible individuals, ciliary dysfunction clubbed with deficient innate mucosal immunity and metaplastic mucosa leads to recurrent and chronic infection of the paranasal sinuses [5,13,17]. Recurrent infections warrant recurrent usage of antimicrobials which often leads to antimicrobial resistance and promotes polymicrobial infections, including fungal infections. Hence. most cases end up in chronic allergic rhinosinusitis [5,7,10]. Most drugs used for symptomatic treatment develop tachyphylaxis with regular usage, warranting a change of drugs or an increase in their dosage. Chronic and recurrent usage of multiple drugs can also lead to adverse effects in many other organs of the body apart from the nasal cavity such as the ocular, hepatic, renal, endocrinal, and gastrointestinal systems [8,9]. Multiple and chronic episodes of sinusitis require multiple surgical interventions to clear the pathways of natural drainage [5,7]. Antral lavage was a commonly used procedure in the past to wash out infectious material or to collect samples for biopsy or microbiological cultures under local anesthesia. Although it provided immediate pain relief, recurrence was common after the procedure [18]. Correction of deviated nasal septum by septoplasty and reduction of enlarged turbinates and widening the osteomeatal complex using endoscopic procedures such as FESS and balloon septoplasty are commonly performed procedures [3,5,11,12,19]. Despite the advancements in management, disease control rates may not be promising at times owing to the multifactorial origin of the disease process [3,5]. Having undergone multiple surgeries and having used multiple treatment options without any benefit, I had resorted to using a modified version of Jalanetikriya, an ancient Indian methodology of cleaning the nasal cavity and sinuses using saline nasal douching upon the advice of an ENT surgeon [14]. Luckily, daily saline nasal douching along with regularly used antihistamines worked well and kept me away from any further episodes of sinusitis for the last three years, giving me a symptom-free interval after 25 long years of immense suffering. Daily saline nasal douching possibly worked by liquifying the thick mucus secretions and washing away the allergens and infectious material on regular basis leaving no scope for colonization and infection. It might have worked as a local antiseptic and promoted effective ciliary motility. Saline nasal douching is an easily available and affordable domiciliary treatment modality without any side effect for the effective management of chronic allergic rhinosinusitis [11,13,14,20].

## Conclusions

Chronic allergic rhinosinusitis is a difficult condition to treat because of its multifactorial etiology. Medical and surgical strategies often fail to appropriately manage this condition in a few cases. Adjunctive daily saline nasal douching is a safe and effective noninvasive domiciliary treatment option for the management of allergic rhinosinusitis.
